# Consensus Report and Recommendations on the Management of Late-stage Internal Derangement of the Temporomandibular Joint

**DOI:** 10.3390/jcm13113319

**Published:** 2024-06-04

**Authors:** Florencio Monje Gil, Patricia Martínez Artal, Alberto Cuevas Queipo de Llano, Mario Muñoz Guerra, David González Ballester, José María López Arcas, José Luis López Cedrún, José Luis Gutiérrez Pérez, Rafael Martín-Granizo, José Luis del Castillo Pardo de Vera, Blas García Medina, Raúl González-García, Manuel Moreno Sánchez, Ekaitz Valle Rodríguez, Jacinto Fernández Sanromán, Ricardo López Martos, Beatriz Peral Cagigal, Marta Redondo Alamillos, Miguel Morey Mas, Carlos Salcedo Gil, Benito Ramos Medina, Adaia Valls Ontañón, Jorge Masià Gridilla, Alicia Dean Ferrer

**Affiliations:** 1 Oral and Maxillofacial Surgery Department, University Hospital Badajoz, 06080 Badajoz, Spain; albertocuevas91@gmail.com (A.C.Q.d.L.); dgonzalezballester@gmail.com (D.G.B.); manumorenosanchez@hotmail.com (M.M.S.); 2 Oral and Maxillofacial Surgery Department, La Princesa University Hospital, 28006 Madrid, Spain; maxmferm@gmail.com (M.M.G.); raulmaxilo@gmail.com (R.G.-G.); 3 Radiology Department, Infanta Leonor Hospital, 28032 Madrid, Spain; jmloparc78@gmail.com; 4 Oral and Maxillofacial Surgery Department, University Hospital A Coruña, 15008 A Coruña, Spain; lopezcedrun@centromaxilofacial.com; 5 Oral and Maxillofacial Surgery Department, University Hospital Virgen del Rocío, 41013 Sevilla, Spain; jlgp@us.es (J.L.G.P.); drlopezmartos@gmail.com (R.L.M.); 6 Oral and Maxillofacial Surgery Department, Hospital Clínico San Carlos, 28040 Madrid, Spain; rmartin.hcsc@salud.madrid.org; 7 Oral and Maxillofacial Surgery Department, La Paz University Hospital, 28046 Madrid, Spain; jldelcastillopardo@gmail.com; 8 Oral and Maxillofacial Surgery Department, University Hospital Virgen de las Nieves, 18014 Granada, Spain; blasmaxilo@yahoo.es; 9 Oral and Maxillofacial Surgery Department, University Hospital Reina Sofía, 30003 Murcia, Spain; ekaitzvalle@hotmail.com; 10 Oral and Maxillofacial Surgery Department, Hospital Povisa, 36211 Vigo, Spain; docvigo@me.com; 11 Oral and Maxillofacial Surgery Department, University Hospital Río Hortega, 47012 Valladolid, Spain; beaperal77@yahoo.es; 12 Oral and Maxillofacial Surgery Department, University Hospital 12 de Octubre, 28041 Madrid, Spain; martaredondo3@hotmail.es; 13 GBCOM Dental and Maxillofacial Clinic, 07014 Palma de Mallorca, Spain; mmoreym@gmail.com; 14 Oral and Maxillofacial Surgery Department, University Hospital Son Espases, 07120 Palma de Mallorca, Spain; 15 Oral and Maxillofacial Surgery Department, University Hospital Santa Lucía, 30202 Cartagena, Spain; drramosmaxilo@gmail.com; 16 Oral and Maxillofacial Surgery Department, Hospital Sant Joan de Déu, 08950 Barcelona, Spain; avalls@uic.es; 17 Oral and Maxillofacial Surgery Department, University Hospital Vall d’Hebron, 08035 Barcelona, Spain; jorgemasiag@gmail.com; 18 Oral and Maxillofacial Surgery Department, Hospital Reina Sofía, 14004 Córdoba, Spain; adeanferrer@yahoo.es

**Keywords:** temporomandibular joint disorders, temporomandibular joint disk, temporomandibular joint diagnosis and temporomandibular joint surgery

## Abstract

**Introduction:** This report investigates late-stage internal derangement (ID) of the temporomandibular joint (TMJ) with the aim of establishing a more effective and personalized treatment protocol to improve patients’ quality of life (QoL). **Material and methods:** A consensus was reached among maxillofacial surgeons specializing in LSID, based on a literature research and collective expert experience following the Delphi method. Consensus was considered to be achieved when a response received at least 80% of votes. **Results:** Four expert groups were established, respectively, focusing on diagnosis, minimally invasive surgery (MIS), open surgery and joint replacement. A comprehensive approach to late-stage ID of the TMJ requires a consensus report. This underscores the need for a personalized treatment plan, considering the variability in clinical presentations and progression of this pathology. Our recommendations aim to optimize clinical outcomes and enhance patient QoL.

## 1. Introduction

Temporomandibular dysfunction (TMD) syndrome encompasses a heterogeneous array of clinical entities affecting the temporomandibular joint (TMJ), masticatory muscles, and adjacent structures. It represents a complex and multifactorial clinical condition, the etiology and pathogenesis of which are not yet fully understood. TMD manifestations commonly include restricted range of motion and TMJ noises, with chronic pain being the primary motivator for patients seeking treatment [1,2].

The global incidence of TMD in the population stands at 34%, with individuals aged 18–60 years being most susceptible. Across continents, it has been observed that the female population comprises 9% to 56% more cases than males [3]. The most common cause of TMJ dysfunction is internal derangement of the joint [4].

Late-stage internal derangement (ID) of the temporomandibular joint (TMJ) is characterized by disc displacement, with or without reduction, resulting in mouth opening limitation. Typically, the articular disc displaces anteromedially, conforming to the condyle shape and the anterior slope of the glenoid fossa, influenced by the lateral pterygoid muscle [4,5]. However, when the mandibular condyle fails to pass over the posterior band of the articular disc, reduction fails, leading to limited mouth opening [5]. Dynamic alterations in TMJ structures may result in morphological consequences such as adherences, disc perforation, and synovial inflammation.

The positional alteration of the joint disc may predispose to mechanical disorders, although it is not always the primary cause of functional deficits. Various factors, including traumatic injury, primary arthritis, hormonal influences, highly reactive oxidative radicals from bruxism, and inadequate lubrication, can contribute to disc derangement by compromising the functional integrity of TMJ ligaments [5].

A broad range of treatment options are available for patients with these disorders, including conservative management, minimally invasive surgery (MIS) and open surgery. Nevertheless, despite these multiple therapeutic options, no clear clinical protocol has been established capable of affording a standardized treatment algorithm adapted to the needs of each individual patient.

The present consensus report seeks to investigate the currently available therapeutic options in depth, and to discuss future perspectives for improving and optimizing the clinical approach to this disease condition. The document aims to establish the bases for a more effective and personalized surgical treatment protocol capable of improving the quality of life (QoL) of patients with late-stage ID.

## 2. Materials and Methods

### 2.1. The Delphi Method

A meticulous protocol following the Delphi method has been followed to reach consensus among maxillofacial surgeons on the treatment of late-stage internal temporomandibular joint (TMJ) lesions. The 24 authors are part of the Spanish Society of Oral and Maxillofacial Surgery and also belong to a group of surgeons with prestigious experience in the field of TMJ, not only because of their published articles on the subject but also their experience and extensive participation in other TMJ conferences. The main characteristics of the Delphi methodology are the following: (1) in an iterative process, participants may express their opinions on several occasions and are offered the opportunity to reflect on these with reference to the majority opinion; (2) anonymity is ensured, decreasing the risk of prestige or leadership bias; and (3) development of the process is controlled by the Delphi coordinators. To address the different aspects, the experts were divided into four groups according to their areas of specialization: diagnosis of late-stage TMJ disorders, minimally invasive surgery (MIS), open surgery, and joint replacement. Our Delphi process was conducted in several rounds, using a series of questionnaires delivered via electronic survey, followed by a meeting of all committee members, in which the findings were discussed, and the recommendations were established to reach the general and well-founded consensus described in detail below.

### 2.2. Study Design

In the initial round, the 24 participants—consisting of oral and maxillofacial surgeons that belong to the Spanish Society of Oral and Maxillofacial Surgery with renowned expertise in TMJ disorders—were asked to recommend diagnostic imaging tools for evaluating suspected advanced internal derangement of the TMJ, and to comment on the correlation between radiological signs and arthroscopic findings in these patients.

Subsequent rounds involved refining consensus items based on the responses received, with iterations focusing on areas of disagreement or uncertainty. The Delphi panelists were presented with the summary of the consensus from the previous round and were asked to re-evaluate their earlier responses in light of the group’s feedback.

Throughout the Delphi process, anonymity of individual responses was maintained to prevent dominance by any one opinion leader and to minimize the bandwagon effect. The iterative nature of the enquiry and controlled feedback allowed the group to work towards the desired consensus.

To finish, the last round was compounded by a meeting of all committee members, in which the findings were discussed, and the recommendations were established to reach the general and well-founded consensus described in detail below. Strong consensus was considered when >95% of participants agreed, consensus was considered when 80–95% of participants were in agreement, and reduced consensus for responses receiving 70–80% of votes. When a response received fewer than 70% of votes, we considered consensus not to have been reached [Table 1].

## 3. Diagnosis of Late-Stage Temporomandibular Joint Dysfunction: Analysis of the Literature and Group Consensus

### 3.1. What Are the Main Symptoms of Late-Stage ID of TMJ?

The main symptom in this stage of the disease is preauricular pain of variable intensity. The pain is typically intermittent and worsens with mandibular function and chewing. Other associated symptoms eventually may also appear, such as otological manifestations (otalgia and occasionally fullness sensation in the ear, manifesting on mobilizing the jaw, and which may be accompanied by dizziness), headache (with specific irradiation towards the maxillary region, temporal zone and frontal area), neck pain (typically with specific trigger points that produce intense pain), and pain in the orbital region (“heavy” and constant pain sensation) [1,6,7,8,9,10].

### 3.2. What Are the Main Exploration Signs in Late-stage ID of TMJ [11,12]?

- Reduced oral aperture, of variable degree and characterized by deviation towards the affected side.
- Normal movement in the ipsilateral direction, but with restriction in the contralateral direction.
- The mechanical alterations in the late stages are usually characterized by the presence of crackling or crepitus, and sometimes the absence of clicking sounds. The sounds are perceived during all of the joint movements, though their characteristics may range from subtle to severe intermittent crackling.
- Pain is produced in response to palpation of the pre-tragus zone with the mouth closed or open. The exploration of this pain is usually completed with the Krogh Poulsen or Mahan bite test: if the pain in late-stage disease is of joint origin, it is triggered on biting upon a thin flat wedge placed in the contralateral molar sector.
- Gross occlusal disorders due to the chronic and progressive evolution of the disorder are much less commonly observed.

### 3.3. What Imaging Techniques Are Indicated in Late-Stage ID of TMJ? What Are the Radiological Findings? Is There a Clear Correlation between the Radiological Signs and the Arthroscopic Findings in Late-Stage Cases?

Panoramic radiography is only of use in ruling out associated disorders in the late stages of joint dysfunction.

When severe dysfunction is suspected, magnetic resonance imaging (MRI) should be considered as first choice, based on T2-weighted sequences (mouth closed–mouth open) in sagittal and coronal views. This technique can evidence the following changes:

- Disc alterations: the joint disc is usually seen to be in a forward position and often shows deformity. Changes in signal intensity are very common in advanced stages.
- Disc perforation: the diagnosis of perforation is difficult to establish and requires great experience on the part of the radiologist, fundamentally using direct or indirect magnetic resonance arthroscopy techniques.
- Joint effusion: this reflects joint inflammation and can be identified in the upper or lower joint spaces, or both.
- Loss of joint space.
- Trabecular bone edema. Formation of subchondral bone cysts.
- Irregularity of the joint contour.
- Formation of exostosis-osteophytes.
- Appositional bone formation of the fossa.

Computerized axial tomography (CAT) or cone-beam computed tomography (CBCT) is clearly useful in distinguishing the joint space and in determining degenerative changes at bone level. These radiological techniques are of use in assessing the following changes:

- Generalized subchondral sclerosis: this is defined as an area of increased cortical bone density extending towards the trabecular component.
- Osteophyte formations.
- Bone erosions.
- Subchondral cysts.
- Flattening of the condylar surface.

Furthermore, magnetic resonance imaging (MRI) is the imaging technique of choice for evaluating the TMJ, due to its capacity to identify soft tissue alterations, disc displacement and joint effusion. However, previous studies have revealed a poor correlation between MRI and arthroscopy, apart from the evaluation of disc reduction and disc perforation.

The current evidence points to a strong correlation between the presence of effusion as evidenced by MRI and the finding of moderate–severe synovitis, with a poorer correlation between disc position and roofing as observed in the arthroscopic exploration. On the other hand, the CBCT findings only seem to be reliable in the determination of joint space collapse and in the assessment of bone structures. Their correlation with the rest of the arthroscopic findings is very limited [13].

In this regard, a reduced consensus was achieved, with 78% agreement among voters that there is a clear correlation between radiological signs and arthroscopic findings in patients with advanced stages of internal derangement of the TMJ.

**Figure 1:**
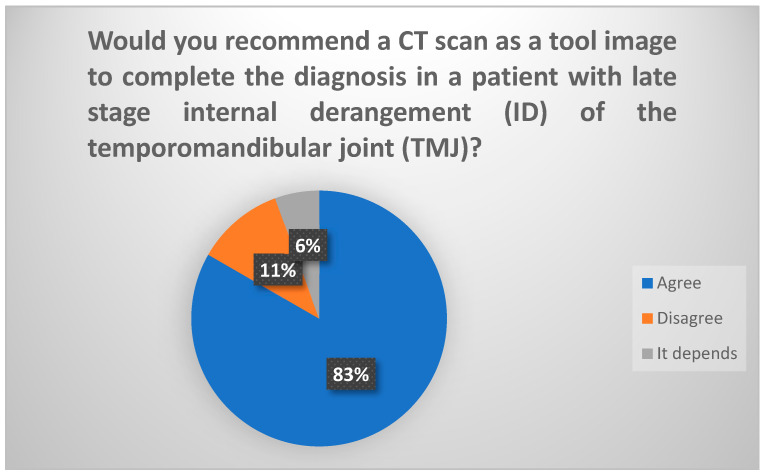
Delphi results regarding use of CT scan in late-stage ID of TMJ.

**Figure 2:**
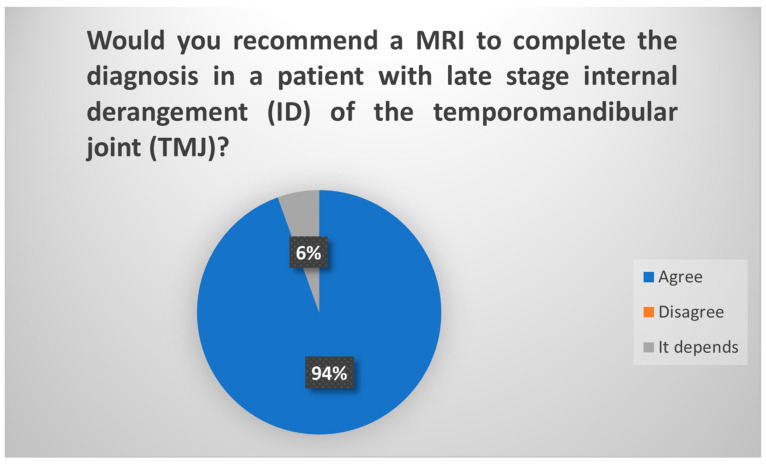
Delphi results regarding use of MRI in late-stage ID of TMJ. Consensus.

According to the findings from the Delphi procedure survey, in the initial round, 71% of participants would recommend CT and MRI as the indicated imaging modalities for a patient suspected of having an advanced stage of internal disorder of the TMJ. However, upon specification in the subsequent round regarding the independent recommendation of CT and MRI, 83.3% of participants would suggest CT [Figure 1] and 94.5% would propose MRI [Figure 2]. Hence, these two tests are deemed optimal in this scenario.

### 3.4. What Are the Possible Arthroscopic Findings in Late-Stage ID of TMJ?

**Figure 3:**
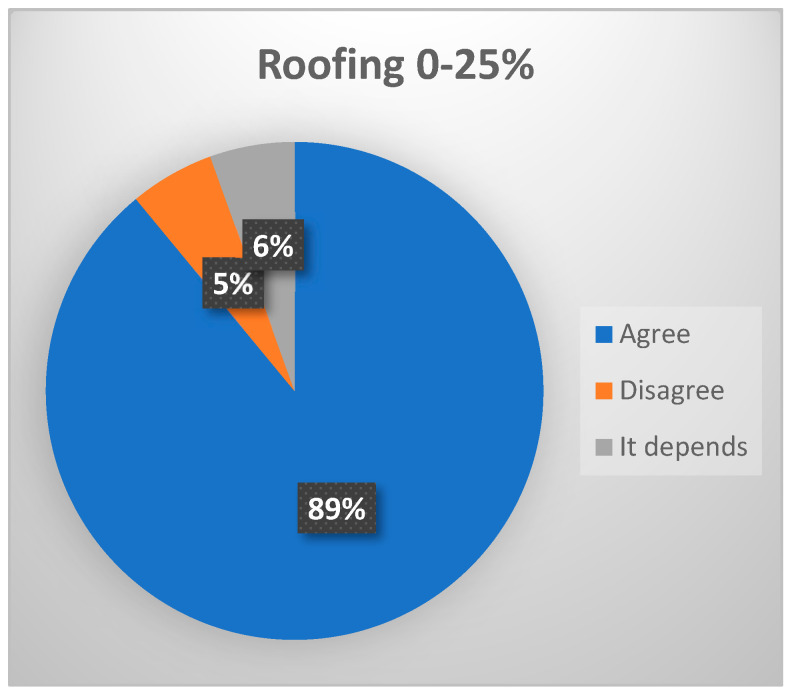
Delphi results: What arthroscopic finding would you expect in a patient with late–stage ID of TMJ? Consensus.

**Figure 4:**
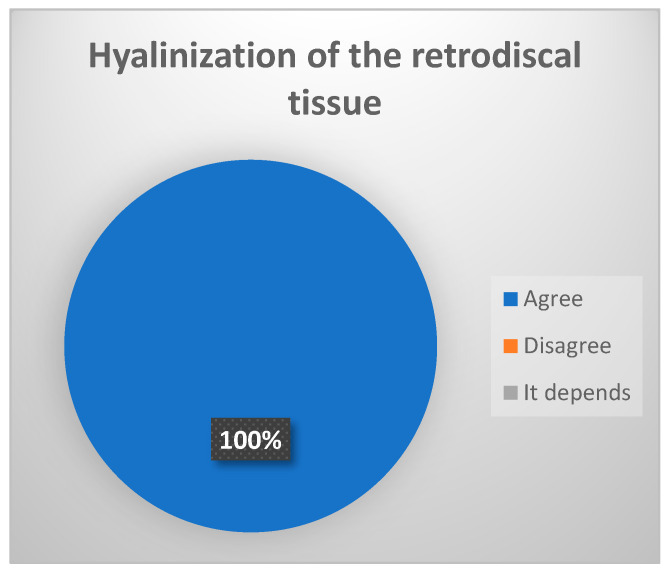
Delphi results: What arthroscopic finding would you expect in a patient with late-stage ID of TMJ? Strong consensus.

**Figure 5:**
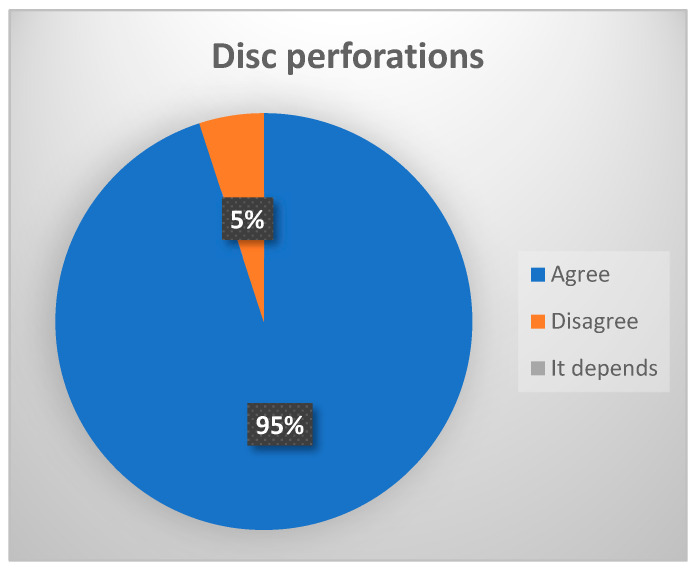
Delphi results: What arthroscopic finding would you expect in a patient with late-stage ID of TMJ? Consensus.

**Figure 6:**
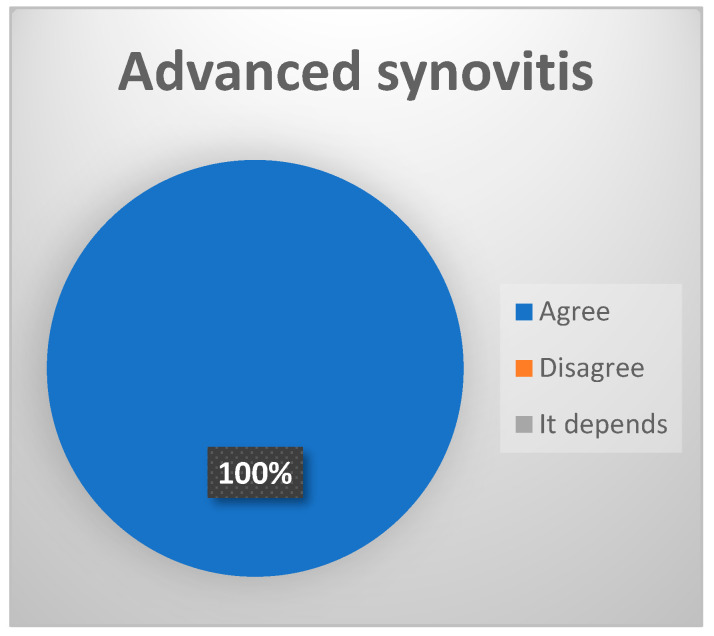
Delphi results: What arthroscopic finding would you expect in a patient with late-stage ID of TMJ? Strong consensus.

**Figure 7:**
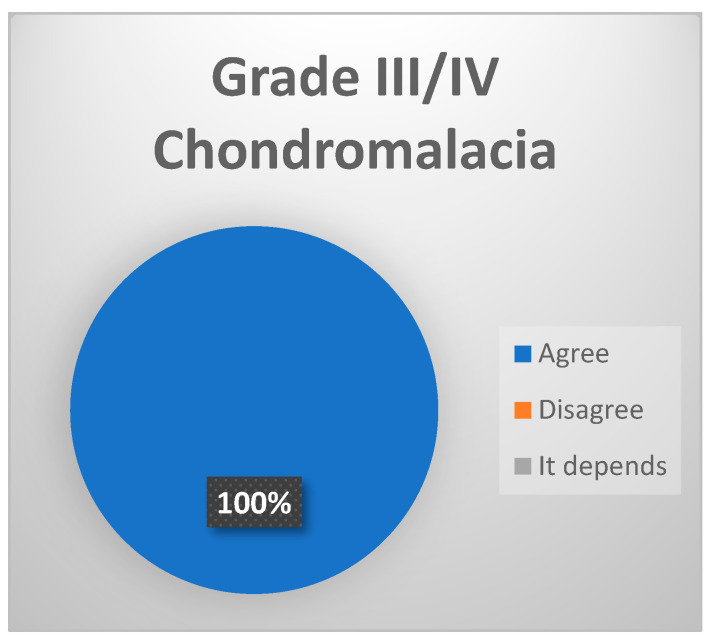
Delphi results: What arthroscopic finding would you expect in a patient with late-stage ID of TMJ? Strong consensus.

**Figure 8:**
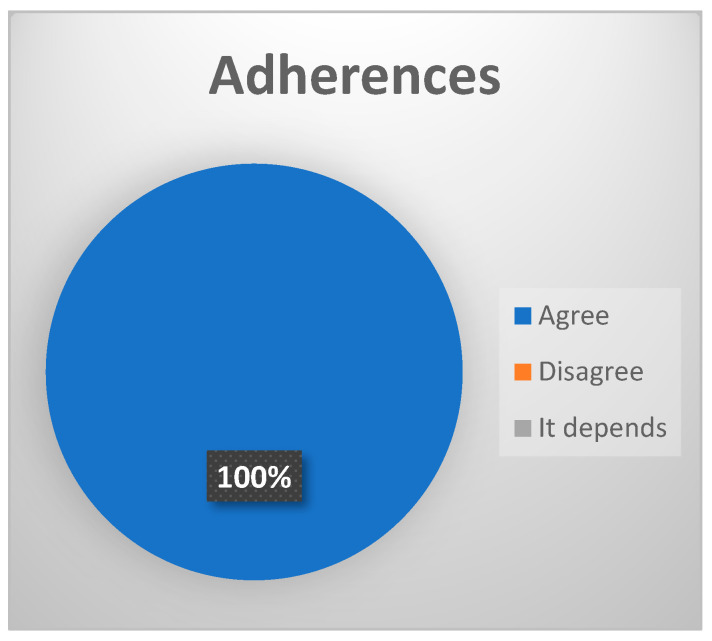
Delphi results: What arthroscopic finding would you expect in a patient with late-stage ID of TMJ? Strong consensus.

The advanced stages of joint dysfunction may show the following morphological changes in the arthroscopic diagnostic evaluation:

- Roofing 0–25%: in cases characterized by disc displacement without reduction (DDwR), “roofing” will be 0% or almost 0%, corroborating the presence of retrodiscal tissue with a variable number of vessels on the surface of the mandibular condyle [14]. (Consensus was reached, with 88.8% of voters agreeing that roofing 0–25% is expected in patients with late-stage ID of TMJ, (Figure 3).)

- Hyalinization of the retrodiscal tissue: metaplasia and remodeling of the bilaminar zone or posterior ligament [14]. (Strong consensus achieved, 100% of votes agree (Figure 4).)

- Disc perforations: These are most often seen at the junction between the retrodiscal area and the joint meniscus, and are usually related to cases of advanced osteoarthrosis [15,16]. (Consensus, 95% of votes agree (Figure 5).)

- Synovial proliferations (“synovial polyps”): these are hyperplastic excrescencies or proliferations of the synovial membrane [17,18,19]. (Reduced consensus, 72.2% of votes agree.)
- Free bone or cartilage bodies: these are fragments of joint cartilage and/or bone that move freely within the joint space [14]. (Reduced consensus 72.2% votes agree).
- Advanced synovitis: inflammation of the synovial membrane. It is most frequently observed in the posterior recess, and according to some studies, its magnitude is correlated with increased intensity of the symptoms [17,18,19]. (Strong consensus, 100% of votes agree [Figure 6]).)
  ○ Grade III and IV of the McCain classification: increased vasodilation, even with possible vascular obliteration, and presenting moderate-to-intense hyperemia; or Grade II of the Holmlund classification: capillary hyperemia and synovial hyperplasia.

- Grade III–IV chondromalacia: softening of the joint cartilage secondary to degeneration produced by joint overload (Strong consensus 100% votes agree, [Figure 7]). In its late stages, we can observe the following:
  ○ Grade III: presence of fibrillation on the joint surface at fossa and eminence level, fading, ulceration and/or rupture of the joint cartilage.
  ○ Grade IV: exposure of subchondral bone.

- Adherences. The following types have been defined: (a) simple fibrous bands; (b) fibrosynovial bands; (c) pseudowalls. Fibrosynovial bands and pseudowalls are more frequent in the advanced stages of the disease [20]. (Strong consensus, 100% of votes agree (Figure 8).)

## 4. Minimally Invasive Surgery in Late-Stage ID of the Temporomandibular Joint: Research and Group Consensus

### 4.1. What Does the Minimally Invasive Treatment of Late-Stage ID of TMJ Consist of?

The treatment of late-stage ID begins with conservative management, with surgery being reserved for those cases that prove refractory to the initial conservative approach. Arthrocentesis (AC) with lavage of the inflammatory and degenerative elements of the TMJ in the advanced stages of the disorder can afford short-term improvement (less than 6 months?) of the pain and joint mobility problems. The infiltration of beneficial substances after arthrocentesis likewise can afford short-term pain and joint mobility improvement compared with isolated infiltrations of the TMJ. These substances may include PRP. The administration of botulinum toxin in cases accompanied by myofascial syndrome in turn contributes to improve any associated surgical technique [21,22].

Level I arthroscopy (lysis and lavage) is effective in diagnosing intraarticular disease, with the added benefits of arthrocentesis. More advanced arthroscopic techniques (levels II and III) are able to act upon the damaged tissues, reducing inflammation, with the sub-synovial infiltration of corticosteroids (CS) or platelet-rich plasma (PRP), the lysis of adhesions, and disc repositioning and stabilization in advanced cases, affording statistically significant improvement especially of the pain and joint mobility [23]. Arthroscopy of the TMJ can delay the need for open surgery in cases of advanced joint dysfunction [24,25].

Repeat arthroscopy in late-stage situations may not be advised, though further studies may focus on this important question due to the paucity of good-quality studies in the high-impact, peer-reviewed literature and more, better-designed studies [21].

### 4.2. Proposal of Minimally Invasive Surgery (MIS) in the Late Stages of Internal Derangement

The proposal of minimally invasive surgery (MIS) in the late stages of internal derangement aims to address the complexities of temporomandibular joint (TMJ) disorders through a progressive treatment approach.

- Initial management of the pain, joint sounds and limitation of mobility of the TMJ: arthrocentesis and joint lavage with the infiltration of PRP and/or hyaluronic acid (HA).
- Addition of botulinum toxin in cases with an associated muscle component (myofascial syndrome).
- Cases refractory to the above treatments: level II arthroscopy of the TMJ with direct therapy targeted to the pathological findings.
- Cases of disc displacement without reduction (recapture) and disc in good condition: level III arthroscopy of the TMJ with associated discopexy is recommended.
- Cases showing poor evolution and in wait of open surgery or joint replacement: corticosteroid infiltrations to reduce the symptoms.

Traditionally, MIS techniques are indicated in cases of joint dysfunction unresponsive to conservative management, provided the patient suffers pain and functional disability with an impact upon his or her daily life. However, there is no clear consensus regarding the appropriate timing of the procedure or the best surgical technique. The data obtained from the radiological study can support the indication of MIS, always in correlation with the clinical findings. Independently of the above, it is advisable to perform an MRI study before any surgery is performed on the joint. Minimally invasive surgery is indicated in patients with a decrease in oral aperture (<35 mm) and/or pain (visual analogue score [VAS] > 3/10), and the clinical data are to be correlated with the radiological findings indicating disc displacement, signs of intraarticular inflammation and the presence of degeneration [14,15].

### 4.3. Infiltration of Different Substances

#### 4.3.1. Are Local Anesthetics a Valid Option in Late-Stage ID of TMJ?

This has not been demonstrated, except for reducing pain over the short term and as part of the surgical management [25]. (Reduced consensus, as 72.2% of votes agree and 27.8% disagree.)

#### 4.3.2. Are Opiates without Side Effects in the TMJ?

**Figure 9:**
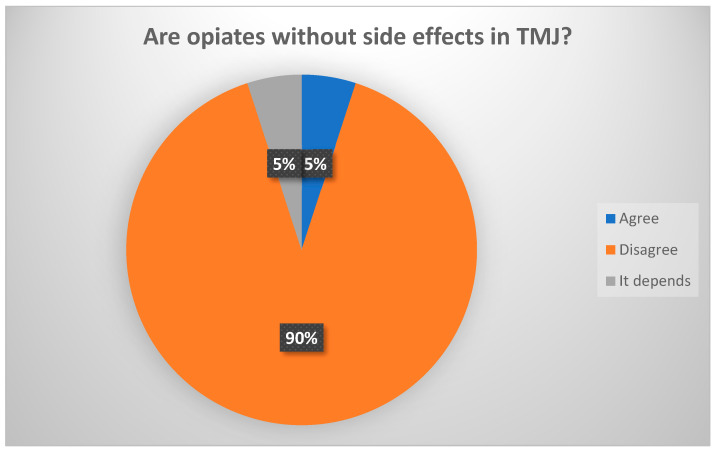
Delphi results: Are opiates without side effects in the TMJ? Strong consensus.

This aspect has not been demonstrated in experimental studies [21]. (Consensus: 89% agreed that opioids have side effects (Figure 9).)

#### 4.3.3. Are Corticosteroids as TMJ Infiltration Therapy Advised?

Corticosteroids (CSs) are indicated in cases of inflammation of the connective (synovial) tissue, the posterior ligament and the medial or anterior capsule (capsulitis) refractory to coblation therapy. They are administered under direct visualization using a long spinal needle (22 G or 27 G) through the skin or the operating portal. In vivo experimental studies indicate that the repeated intraarticular administration of CSs at high doses induces irreversible degenerative tissue phenomena, and this practice is therefore not advised [26]. Corticosteroids are effective in reducing joint pain over the short term (less than 5 months). Intraarticular CS infiltration significantly increases maximum oral aperture (MOA) over the short to middle term (less than 4 years). Such treatment shall be used in very advanced cases requiring future open surgery with joint replacement in order to reduce the joint pain. Although confirmation is lacking, chrono dose depot betamethasone (3 mg), due to its slow-release characteristics, appears to be the most effective option. It has not been shown to exert a synergic effect with other substances, though such treatment can be used in very advanced cases requiring future open surgery with joint replacement to reduce the pain, in combination with other regenerative substances (PRP) [27]. (No consensus was reached, as 66.7% disagreed in advising corticosteroids as TMJ infiltration).

#### 4.3.4. Are There Differences between Infiltrations of the TMJ with Hyaluronic Acid of Different Composition and Origin?

**Figure 10:**
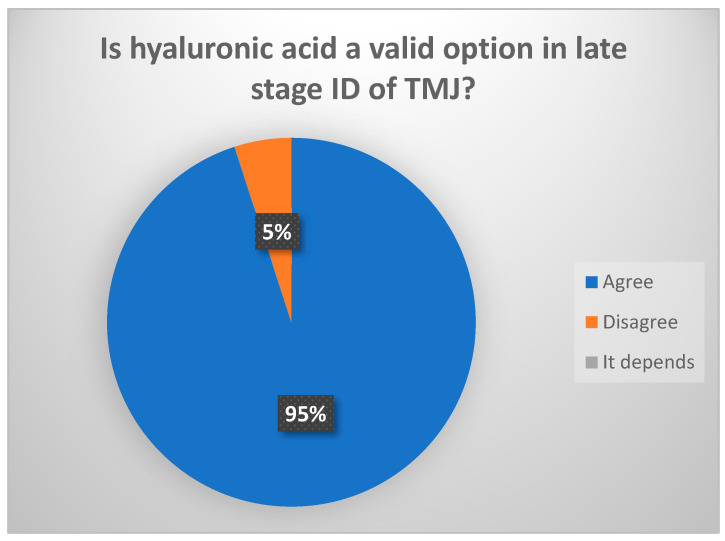
Delphi results: Is hyaluronic acid a valid option in late-stage ID of TMJ? Consensus.

The intraarticular infiltration of hyaluronic acid (HA) after arthroscopy (level I–III) significantly increases MOA over the short to middle term (less than 4 years) (consensus reached: 94.4% of votes agreed, (Figure 10)). Randomized studies have found no differences between different types of HA. It seems that HA exerts an intraarticular occupying effect, avoiding clot formation, though it would also improve tissue healing. High-density HA remains for longer periods in the joint according to data from orthopedic studies. The intraarticular infiltration of HA without lavage significantly reduces pain over the short term (less than 5 months). On the other hand, high molecular weight HA is degraded within a few weeks, though the use of non-animal stabilized hyaluronic acid (NASHA) (lacking antigenic capacity) with gel-like high density is able to persist within the joint for up to 6 months. Clinical trials have reported a decrease in its effect from 6 months onwards. No studies have evaluated the infiltration of HA alone versus HA combined with PRP, though it seems that when both components are mixed in the same tube, intraarticular permanence is increased [28,29].

#### 4.3.5. What Are the Benefits of Platelet-Rich Plasma (PRP) Infiltration in Arthroscopy of the TMJ?

**Figure 11:**
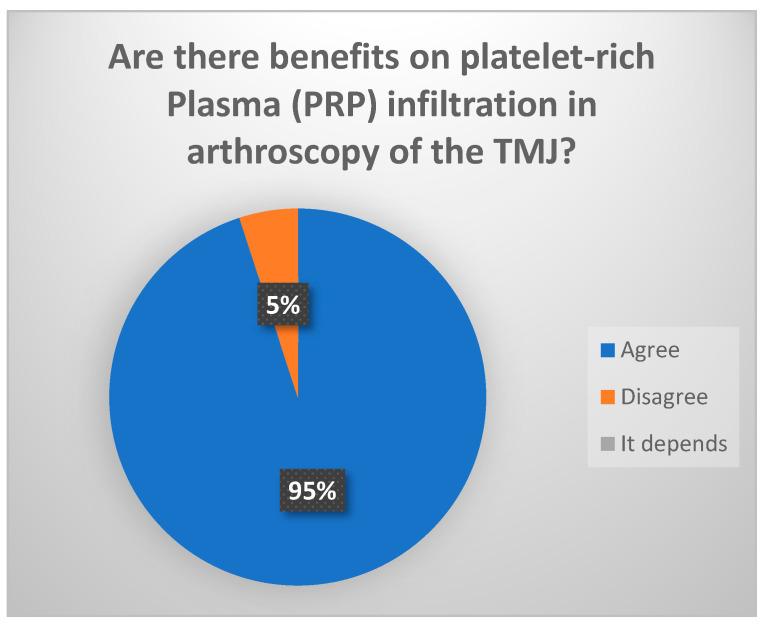
Delphi results: Are there benefits with platelet-rich plasma (PRP) infiltration in arthroscopy of the TMJ?

The intraarticular infiltration of PRP following arthroscopy (level I–III) significantly reduces pain and increases MOA over the short to middle term (less than 4 years). Moreover, it may afford an anti-inflammatory effect and would generate no adverse reactions, since it is of autogenous origin (consensus reached: 94.4% votes agreed (Figure 11)). Comparative studies have reported improved outcomes in terms of pain when applying direct infiltrations of HA, PRP or CS versus infiltrations associated with arthrocentesis or arthroscopy. This could be explained by the repetition of the infiltrative procedures over time [30,31,32,33,34,35,36].

#### 4.3.6. What Procedures Are Advisable in Arthroscopy in Late-stage Disease?

The recommended procedures comprise synovectomy via coblation or laser, the subsynovial infiltration of PRP or CS, biopsies, and the resection of adhesions and pseudowalls [5,37,38,39,40,41,42,43,44,45,46].

## 5. Open Surgery in the Late Stages of ID of TMJ: Research and Group Consensus

### 5.1. What Is the Open Surgery Technique of Choice As a First Alternative to Conservative Management?

Although there are not enough quality studies, the current evidence recommends conservative management as first line treatment in the late stages (stages IV and V of the Wilkes classification) of TMJID. When these measures fail, minimally invasive procedures such as surgical arthroscopy should be the surgical option of choice, and only in those cases where these techniques prove ineffective in controlling pain or the limitation of oral aperture should arthrotomy be considered [5].

If the joint disc can be repositioned, discopexy with fixation of the disc using sutures or bone-anchoring miniscrews could be the best option for preserving joint integrity, though the clinical success rate is low (about 50%) [47,48,49,50,51].

The main indications for disc repositioning are internal derangements with anterior displacement of the disc, without reduction (closed lock), with severely limited oral aperture in patients who have failed to respond to conservative management or MIS in the form of arthrocentesis or arthroscopy [50].

In contrast, if the disc is irreparable (because of major deformations or perforations), with an oral aperture of less than 25 mm, radiological signs of generalized osteoarthrosis, severe degenerative joint changes and intense pain (VAS score 9/10) after arthroscopy, discectomy with or without interpositioned materials would be the most appropriate technique [52].

Following the technique, it is essential to ensure correct condylar motion, without rotation/translation obstruction. Discectomy always should be complete, making sure not to leave any remnant disc in the areas where intraoperative control proves most difficult (medial and anterior zone) [52].

However, the outcomes to be expected from open surgery worsen as the joint degeneration process advances. In most studies, the success rate of the technique does not appear to exceed 70% [51].

### 5.2. When Are Open Surgery Techniques Indicated in the Treatment of Late-Stage Disease?

Open surgeries are only indicated after the failure (persistence of non-manageable pain, chronic joint lock with limitation of oral aperture) of noninvasive surgical techniques such as surgical arthroscopy, or when such techniques are not available.

### 5.3. How Long Is It Necessary to Wait after Failed Arthroscopy in Order to Indicate an Open Surgery Technique?

The period will depend on the clinical course of the patient after a minimum follow-up period of 6 months. (Strong consensus reached: 100% agreed that the waiting period after failed arthroscopy should be between 6 and 12 months.)

### 5.4. What Are the Disc Replacement Materials of Choice after Discectomy?

Following discectomy, the constant contact between the bone surfaces could interfere with the diffusion of synovial fluid nutrients, resulting in remodeling of the joint bone surfaces. In this regard, disc replacement can help prevent or reduce bone remodeling, adherences and recurrent pain [53].

There is no evidence in the literature of differences in the outcomes between discectomy with or without disc interpositioning, though it is estimated that almost one-half of all surgeons currently perform disc replacement. The interpositioned material must be safe, predictable and available in sufficient quality and volume, or be easy to produce [54,55].

Alloplastic materials (Proplast-teflon) are now little used, due to the numerous complications they present. Silicone may be used as a temporary spacer, however [53,54].

On the other hand, autografts classically have been the most widely used option:

- Adipose tissue grafts offer optimum adaptability, as they are easily moldable and afford the highest success rates in long-term studies. In addition, they improve patient quality of life [56,57,58].
- Temporal flaps can include fascia or muscle; they are accessible and cause scant morbidity for the patient [53,54]. However, their predictability over the long term is not clear, and there have been reports of heterotopic ossification with vascular and joint space impairment [59].
- The ear cartilage is morphologically very similar to the cartilage of the joint disc and is easily accessible. However, although short-term results are satisfactory, with a decrease in pain and improved oral aperture, joint degeneration over the long term is not avoided [60].

With regard to allografts, mention must be made of cryopreserved human amniotic membrane (HAM), which affords results comparable to those of a temporal muscle flap [61,62]. However, the re-epithelializing properties of HAM could favor the neoformation of ectopic bone, causing new functional limitation and even ankylosis [63].

Recent studies have explored the potential of tissue engineering as a tool for tissue replacement [64,65].

In relation to the Delphi survey, when asking participants if they would perform disc replacement after discectomy, 55.6% indicated that they do not place a disc substitute, 33.3% sometimes do and 11% always do, although no consensus limit was reached. However, in the second round, when asked if they would place disc replacement, what type of material would be used, 78% would use autograft. There is much controversy regarding this matter in the literature, as occurs in the consensus of the participants.

### 5.5. What Is the Recommended Material in the Event of Joint Disc Replacement?

Alloplastic materials should not be used for permanent disc replacement (strong consensus reached: 100% agreement on the contraindication of alloplastic materials for permanent use). Use can be made of autologous grafts such as adipose tissue (from the abdomen), temporal muscle or cartilage (from the ear or tissue bank), since the interpositioning of these autologous materials appears to result in faster clinical improvement of the patient than when the disc is not replaced [53,54].

Consideration is required of the possible complications and morbidity associated with autologous graft harvesting, and the patient should be adequately informed about the possible risks and benefits [53,54].

### 5.6. Are Other Techniques Complementary to Disc Replacement or Discectomy Indicated [52,64,65]?

Discopexy or discectomy may be complemented with eminoplasty and condyloplasty or condylar shaving. These techniques are indicated in the presence of osteophytes or other degenerative joint changes, or when the eminence is very large and complicates translation of the condyle-disc complex. The existing joint cartilage should always be preserved in order to avoid progressive joint degeneration; for this reason, the above treatments are currently very little used [54,66,67].

### 5.7. What Clinical Results Can Be Expected after Open Surgery of the TMJ?

Between 50–70% of all operated patients will not need further surgeries, though nevertheless, there may be some persistent joint dysfunction or postoperative radiological findings consistent with adaptive joint changes [67].

### 5.8. What Should Be Done after Failure of Any of the Described Surgical Techniques?

In the event of failure, total joint replacement should be indicated, and according to the reviewed literature, this may occur in 30–40% of the cases. It is important to avoid increasing the number of prior surgeries as far as possible, in order not to reduce the efficacy of joint replacement and increase future morbidity for the patient [68].

## 6. Joint Replacement in the Late Stages of Temporomandibular Joint Internal Derangement: Research and Group Consensus

### 6.1. Indications for Joint Replacement

#### 6.1.1. What Are the Clinical Criteria for Considering Joint Replacement in Late-Stage Disease?

Candidates for joint replacement are patients with a pain score of 50/100, reduced oral aperture (less than 35 mm) and problems with chewing solid food (diet score of less than 50/100) who fail to respond to other therapeutic measures. The radiological studies (CBCT and/or MRI) evidence alteration and degeneration of the joint structures at both bone (osteophytes, geodes, subchondral cysts) and disc level [50,69,70].

Joint replacement should be regarded as the last treatment option when conservative management, MIS or open surgery have failed [69].

#### 6.1.2. What Prognostic Factors Have a Negative Impact upon the Outcome of Joint Replacement?

The prognostic factors with a negative impact upon the outcome are severe preoperative pain, a large number of prior surgeries, and an important use of opioids. These factors indicate that joint replacement in the late stages of joint dysfunction, when the other treatments have failed, should be performed [71].

#### 6.1.3. Is There a Maximum Number of Prior Surgeries before Considering Joint Replacement?

The data published by Mercuri indicate that patients subjected to 0–2 previous arthrotomies present a success rate of 75% after alloplastic replacement. In those with 3–5 previous arthrotomies, the figure drops to 50%, and in patients with more than 6 previous surgeries, the percentage success rate is 25%. Therefore, only a single arthrotomy (if it is an appropriate procedure performed by an experienced surgeon) should be performed, knowing that 39% of those patients subjected to this technique will have an unfavorable outcome, and joint replacement may need to be considered [72].

#### 6.1.4. Does Concomitant Disease (Collagen Diseases, Fibromyalgia) Have a Negative Impact upon the Outcome?

The pathophysiology of inflammatory diseases of the joints is very different from that of degenerative disease of the joints. Only limited data can be found in the literature regarding joint replacement in patients with a history of inflammatory joint disease such as rheumatoid arthritis, ankylosing spondylitis, psoriatic arthritis, juvenile idiopathic arthritis or systemic lupus erythematosus, though the results published to date have been favorable [73].

However, it must be taken into account that these patients typically receive immune suppressors such as corticosteroids and disease-modifying antirheumatic drugs. These treatments can cause a delay in healing and facilitate wound infection during the postoperative period. It is therefore advisable to consult the rheumatologist in such cases to see if it is possible to suspend these drugs for a period of time [73].

#### 6.1.5. In Patients with Bilateral Late-stage Disease, Should Joint Replacement Be Bilateral or Limited Only to the Symptomatic Joint?

When there is no disease of the contralateral joint, the latter should not be replaced (consensus reached: 83.4% of votes agree). However, when there has been some prior or concomitant surgery of the contralateral TMJ, 30% of the patients may finally require total joint replacement in the future. On the other hand, when replacement of both joints is indicated, it is advisable to perform treatment bilaterally in the same surgery [74,75].

#### 6.1.6. Does Patient Quality of Life Improve after Alloplastic Replacement of the TMJ?

Patient quality of life (QoL) improves substantially in the following terms: improved joint function and range of oral aperture (if restricted to <30 mm before TMJ replacement), with better chewing and eating capacity, and control of pain at both joint and facial level. Improvement has also been reported in terms of activities of daily living, leisure activities and mood, with a decrease in anxiety scores. Furthermore, the findings in terms of duration and biocompatibility obtained from long-term implant follow-up studies define this treatment as the option of choice when deciding to replace a joint in the late stages of dysfunction. In this respect, it is very advisable to administer a quality of life questionnaire among those patients who are going to undergo alloplastic joint replacement [76,77,78].

### 6.2. Controversies of the Surgical Technique

#### 6.2.1. What Is the Joint Replacement Technique of Choice in Late-Stage Dysfunction?

The joint replacement options include osteogenic distraction, autologous grafts and implants. In terms of long-term stability and quality of life, most studies advocate alloplastic replacement as the technique of choice for reconstruction of the TMJ.

#### 6.2.2. Are Stock Implants Indicated in Late-Stage Disease?

Both stock and custom implants offer good results, with improvement of maximum interincisal aperture, pain reduction, improved diet quality, and better quality of life. The main inconvenience of stock implants is their adaptation problems in situations characterized by important anatomical alterations. Virtual surgical planning and the manufacture of cutting guides therefore offer greater predictability in cases where stock implants are to be used [79].

Thus, the use of stock implants may be considered except in those cases in which they are clearly not advisable. In turn, the indications for custom implants are the following [80,81,82]:

- When a gap of >35 mm is created between the fossa and the mandibular ramus.
- In the presence of important anatomical alterations, as in the case of ankylosis or tumor resections.
- Syndromic patients.
- When combining concomitant orthognathic surgery.
- Joints that have been operated upon a number of times, particularly in the case of replacements of other joint implants.

#### 6.2.3. New Designs Such as Implants with Replacement of the Fossa and Others: Are They Useful?

New implants have been developed with the aim of overcoming a number of problems that have still not been resolved with the conventional designs:

- Replacement of one of the components of the fossa.
- Wings or tabs in the condylar fragment that contour the posterior and inferior margin of the mandible, to facilitate placement and improve precision in location of the condylar fragment.
- Wire anchoring orifices between the condylar fragment and the component of the fossa, to control or limit excessive condylar excursions.
- Reinsertion of the lateral pterygoid muscle.

The evidence on the effectiveness of all of these designs will be established in the future through the publication of patient case series comparing the different implant designs.

#### 6.2.4. Is Reinsertion of the Lateral Pterygoid Muscle Useful?

Recent mandibular kinematic studies attribute the limitation of laterality and protrusion movements to deinsertion of the lateral pterygoid muscle on performing the condylectomy. At present, new mandibular implant designs are being investigated that include a scaffold to favor lateral pterygoid muscle reinsertion and enthesis. Few studies confirming their usefulness have been published, however [83,84].

#### 6.2.5. Is Adipose Tissue Grafting for Covering the Implant Indicated?

The use of autologous adipose tissue grafts from the abdomen to cover the alloplastic TMJ implant aims to obliterate dead spaces and limit hematoma formation. This may reduce the probability of heterotopic ossification by up to 20%. It is indicated in cases characterized by a high risk of ankylosis [85,86]. (No consensus was reached: 61.1% indicated adipose tissue grafting only in cases of TMJ ankyloses and 27.8% always perform this technique.)

#### 6.2.6. Is Coronoidectomy in Joint Replacement Indicated?

Coronoidectomy is usually required in patients with ankylosis, severely limited oral aperture, or interference with the zygomatic arch. However, in cases where a lack of stability of the TMJ is expected, coronoidectomy may increase the instability. Therefore, in patients with late-stage TMD, coronoidectomy is not systematically indicated [87].

#### 6.2.7. Navigation and Guided Surgery

Surgical navigation improves the precision of the results of joint replacement and has been shown to be useful for the following [88,89]:

- Performing complete, precise and safe resection of the glenoid cavity, where indicated.
- Avoiding damage to important structures during surgery.
- Increasing safety and reducing uncertainty during the surgical procedure.
- Checking the results of placing both implant components intraoperatively and performing any necessary corrections.
- Designing trajectories for precise placement of the implant screws.
- Performing virtual surgery preoperatively by entering both stock and custom implants in the navigator planning software.

## 7. Conclusions

The consensus report on late-stage temporomandibular joint dysfunction emphasizes the need for a personalized, multi-disciplinary approach to diagnosis and treatment. Accurate diagnosis using MRI and CAT/CBCT is crucial, guiding a treatment progression from conservative methods to more invasive surgeries as needed. The report highlights the significance of patient-specific strategies, including minimally invasive and open surgeries, and considers joint replacement as a final option to enhance quality of life. It underscores the importance of specialist collaboration in managing complex cases and advocates for ongoing research to refine treatment methods, ultimately aiming to improve outcomes and quality of life for patients with late-stage TMD.

## Figures and Tables

**Table 1:** Strength level of consensus.

Strong consensus	>95% of participants agreed
Consensus	80–95% of participants agreed
Reduced consensus	70–79% of participants agreed
No consensus	<70% of participants agreed

## Data Availability

The data presented in this study are available on request from the corresponding authors.
